# Designing and Preparation of Fiber-Reinforced Composites with Enhanced Interface Adhesion

**DOI:** 10.3390/polym10101128

**Published:** 2018-10-11

**Authors:** Dengxun Ren, Lin Chen, Yue Yuan, Kui Li, Mingzhen Xu, Xiaobo Liu

**Affiliations:** Research Branch of Advanced Functional Materials, School of Materials and Energy, University of Electronic Science and Technology of China, Chengdu 61173, China; rendenxun2008@126.com (D.R.); linchen_uestc@163.com (L.C.); moonsunmars@163.com (Y.Y.); leekingue2014@163.com (K.L.)

**Keywords:** interfacial adhesion, surface modification, copolymerization, physical entanglement

## Abstract

The interfacial properties between fibers and resin matrices show great influence on the properties of fiber-reinforced composites. In this work, phthalonitrile containing benzoxazine (BA-ph) was chosen as the resin matrix, which combined with the glass fiber (GF) to prepare reinforced composite laminates at low temperature (200 °C). The poly(arylene ether nitrile) (PEN) was used to modify the GF and BA-ph matrix. Curing behaviors of the BA-ph/PEN were investigated with Differential scanning calorimetric (DSC) and Dynamic rheological analysis (DRA), and results indicated that the polymerization would be hindered by PEN due to the dilution effects. Moreover, the formation of triazine rings which assigning to the ring-forming polymerization of nitrile groups in BA-ph and PEN could improve the compatibility of BA-ph and PEN in the matrix. The SEM images of the fracture surface of the composites revealed that the brittleness of BA-ph matrix and interfacial adhesion between GFs and matrix was improved. The enhanced interfacial adhesion was detailedly discussed from the perspective of physical entanglement and the copolymerization between PEN chains on the surface of GFs and BA-ph/PEN matrix. The results of DMA also explained the toughness of BA-ph/PEN matrix, the semi-interpenetrating polymer networks and the interfacial adhesion. In sum, a feasible strategy that modifies the surface of GFs and the brittleness of the thermosetting matrix by high-performance thermoplastic polymers, which can be employed to prepare the composite laminates with improved properties.

## 1. Introduction

With the development of economy and science, the demand of high performance polymer-based composites is rapidly increased over the past years. In the areas such as automobile, electronics, new energy, and especially for the aeronautics-aerospace industry, the fiber reinforced polymer composites were widely used as the substrate and structural materials. Compared to the traditional metal materials, fiber reinforced matrix composites possess the properties such as light weight, good acid and alkali corrosion resistance, easy processing, low moisture absorption, low dielectric constant and loss, good creep resistance and thermal ability, therefore the application of high-performance matrix composites in the fields mentioned above increased and also can lower the costs, simultaneously [1,2,3,4,5,6,7,8]. Phthalonitrile resins, as one of the high-performance thermosetting resins, had drawn much attention on the researches and applications of advanced fiber-reinforced composites due to their outstanding flexural strength (≥500 MPa), high thermal-oxidative stability (*T_5%_* ≥ 350 °C), excellent flam resistance, high Young’s modulus (≥20 GPa) and low water absorptivity [9,10,11,12]. However, the difficulty of their polymerization has limited the wide application of the phthalonitrile-based resins and their composites. Among them, phthalonitrile containing benzoxazine (BA-ph), due to its autocatalytic polymerization, has been applied widely. Assigning to the active hydrogen and amine structures generated from the ring-opening polymerization of benzoxazine rings, the nitrile groups would be effectively catalyzed and ring-forming polymerization of nitrile occurred, consecutively [13]. The previous work indicated that the fiber-reinforced composites based on BA-ph exhibited satisfactory mechanical properties and excellent thermal stability [3,12,14,15]. However, the common defects including the weak interfacial adhesion between the matrix and the fibers, the local resin-rich inside the composites and the brittleness of the matrix, existed in the fiber-reinforced BA-ph composites. Thus, in order to obtain improved phthalonitrile-based fiber-reinforced composites, studies on the designing and preparing of fiber-reinforced matrix composites are necessary.

As is well known, the fibers bonded by matrices in the fiber-reinforced composites play the roles that carry the load and hinder the crack initiation and propagation when the composites under the load [16,17]. Thus, the strength of the matrices and the interfacial adhesion between fibers and matrices would mainly determine the final properties of the composites. As previously reported, the interface adhesion between fibers and the matrices could dominate the properties of the composite laminates, especially the mechanical properties [18,19,20,21,22]. Therefore, studies on the improvement of the interface adhesion between fibers and matrices have been carried out recent years. The glass fiber modified by titanate couplers showed outstanding interfacial adhesion with the polymer reported by Jang et al. [23]. Jing and the coauthors reported the comparison study on glass fiber modification that the glass fiber treated by silane coupling agents and graphene oxide, respectively. Results indicated that the glass fiber coated with graphene oxide significantly improved the mechanical properties and remained relatively better toughness [24]. Other researchers reported novel fiber modification method to obtain a modified fiber reinforced composites by ex-situ modification. That is, introducing a film former on the surface of the fiber before it was infused with the matrices. Results indicated that the ex-situ modification can provide improved interfacial adhesion between the glass fiber and matrix [25]. In sum, the modifications on the surface of the fibers with polymers and inorganic nanoparticles could greatly improve the properties of fiber-reinforced composites. However, for the modification with inorganic nanoparticles, the treatment should be realized under the harsh experimental conditions and the costs may be increased with complex modification operation; for the modification with polymers such as silane coupling agents, the surface modification may not meet the requirements during the processing with high temperatures. Thus, the studies on the novel modification methods of the fiber-reinforced composites are urgent need for research. In our previous work, the Poly(arylene ether nitrile) (PEN), a kind of excellent engineering thermoplastic with excellent properties, such as high glass transition temperature (*T_g_* > 160 °C), good mechanical properties, high thermal stability (*T_5%_* > 370 °C) [26,27,28] and good solubleness, was introduced into the phthalonitrile-based composites to obtain the improved fiber-reinforced composites [18]. Results demonstrated that the composites with the PEN decorated on the surface of the fibers possess more outstanding mechanical properties, indicating the surface modification of fibers with PEN could significantly promote the performance of composites. Moreover, with the introduction of the PEN, the mechanical properties of the composites improved in comparison with that of the pristine phthalonitrile-based composites. However, the detailed improvement mechanism of the PEN on the composites was not discussed and revealed.

In this work, the detailed effects of the PEN on the interfacial adhesion and final properties of the fiber-reinforced BA-ph composites were studied. Based on the previous results, the PEN was first applied to modify the surface of the glass fibers. Then, various contents of PEN were introduced into the BA-ph matrix to fabricate the modified fiber-reinforced composites. To further understanding the effects of PEN on the polymerization of BA-ph and the interface adhesion between fibers and BA-ph, the curing behaviors, process properties and fracture surface were investigated by differential scanning calorimetric (DSC), dynamic rheological analysis (DRA) and scanning electron microscope (SEM), respectively. Thermal-mechanical properties and the thermal stability of the composites were also studied to confirm the improved properties.

## 2. Experimental

### 2.1. Materials

The phthalonitrile containing benzoxazine (BA-ph, yellow powder, softening point temperature 86 ± 2 °C) [29] and bisphenol A based Poly(arylene ether nitrile) (PEN, white powder, glass transition temperature *T_g_* = 178 °C) [26] were prepared in our laboratory. 1-Methyl-2-pyrrolidinone (NMP, purity 99%, AR) was purchased from Kelong reagent Co., Ltd., Chengdu, China. The glass fiber (GF, EW170-100) fabric was obtained from Shenyang Gaote glass fiber Co., Shenyang, China. The PEN was washed in boiling water three times (10 min for each time) and dried in oven (Keelrein Instrument Co., Ltd., Shanghai, China) at 100 °C for 24 h, the GF was treated at 200 °C for 1 h. Other materials were used without further purification.

### 2.2. Preparation of PEN Modified Glass Fibers

PEN was solved in NMP at room temperature to obtain the viscosity PEN solution. The GFs were first heat treated at 200 °C for 1 h and then infused with the above PEN solution. The content of PEN was controlled as 4 wt % according to the weight of GFs. Then, the impregnated GFs were dried in the oven at 80 °C for 10 min, 140 °C for 15 min to obtain the modified GF, labeled as *p*-GF.

### 2.3. Preparation of BA-ph/PEN Blends with Various Content of PEN

Various contents of PEN (0, 10, 20, 30 and 40 wt %, in the mass ratio of BA-ph/PEN) were mixed with BA-ph to prepare the BA-ph/PEN blends. The preparation of BA-ph/PEN blends is as follows: the BA-ph was dissolved in NMP in a three-necked flask (Shu Niu Glass Instrument Co., Ltd., Chengdu, China) equipped with a mechanical stirrer (Changzhou Rong Hua Instrument Manufacturing Co., Ltd., Changzhou, China) at 160 °C for 2 h, then the PEN with NMP were added into the prepared BA-ph solution at 160 °C for 1 h and then the BA-ph/PEN blends were obtained.

### 2.4. Preparation of BA-ph/PEN/p-GF Composite Laminates

The weight fraction of GF and matrix were designed as 60 and 40 wt %, respectively. The *p*-GFs were impregnated with the as prepared viscosity BA-ph/PEN solution. 2 layers of the impregnated *p*-GFs were piled together and put on the flattened clean glass plate in the oven at 80 °C for 4 h, after that the prepregs were suspended in the oven at 160 °C for 10 min to remove the solvent. Finally, the prepared prepregs were placed in a stainless mold (Chengdu Wei Da Machinery Manufacturing Co., Ltd., Chengdu, China) (10 cm × 10 cm × 1.5 mm) under the pressure of 20 MPa via the curing procedure 180 °C/2 h and 200 °C/2 h. The BA-ph/PEN/*p*-GF composite laminates with various contents of PEN in the BA-ph matrix were prepared according to the process above presented.

### 2.5. Characterizations

Differential scanning calorimetric (DSC) analysis was applied to investigate the curing reaction of the blends though TA Instruments Modulated DSC Q100 (TA Instruments, Newcastle, DE, USA) at a heating rate of 10 °C/min under nitrogen atmosphere with the flow ratio 50 mL/min. Dynamic rheological analysis (DRA) was tested by using TA Instruments Rheometer AR-G2 (TA Instruments, Newcastle, DE, USA) with a frequency of 1 Hz at 160, 180 and 200 °C for different time in air atmosphere. The flexural experiments (three-point bending mode) were carried out by a SANS CMT6104 series desktop electromechanical universal testing machine (Shenzhen SANS, Shenzhen, China) at room temperature with crosshead displacement speed of 5 mm/min, and the test fixture was mounted in 10 kN capacity. The samples (40 mm × 15 mm × 1.5 mm) were tested with a support span/sample thickness ratio of 16:1, and obtained the average value for every three samples. The morphology of the fractured surfaces of the composites was scanned though scanning electron microscope (SEM, JSM25900LV, JEOL, Akishima, Japan) operating at 20 kV. Dynamic mechanical analysis (DMA) in the mode of three-point bending with a frequency of 1 Hz was tested on a TA Instruments QDMA-800 (TA Instruments, Newcastle, DE, USA) from 50 to 350 °C at the heating rate of 5 °C /min under air atmosphere. The dimension was 40 mm × 10 mm × 1.5 mm, and the storage modulus and *tan δ* were investigated. Thermogravimetry (TGA) was tested on a TA Instruments TGA Q50 (TA Instruments, Newcastle, DE, USA) with a heating rate of 20 °C/min under nitrogen from 50 to 800 °C.

## 3. Results and Discussion

### 3.1. Curing Behaviors of the BA-ph/PEN Blends

**Figure 1:**
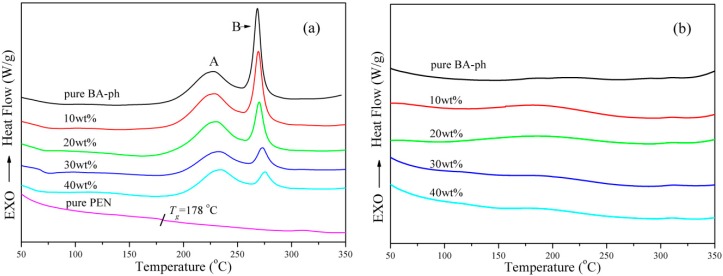
DSC curves of BA-ph/PEN blends, PEN and the cured BA-ph/PEN matrix: (**a**) BA-ph/PEN blends and pure PEN and (**b**) the cured matrix of the BA-ph/PEN blends.

**Figure 2:**
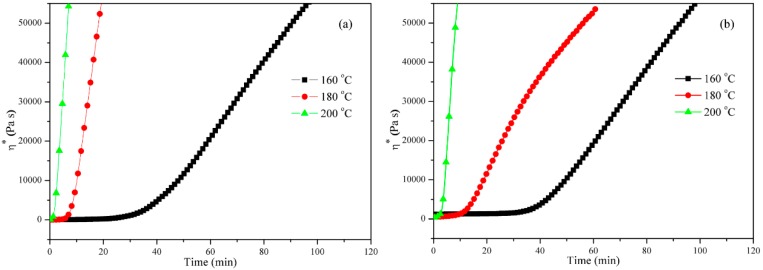
Viscosity of the pure BA-ph and BA-ph/PEN blends with 20 wt % PEN content measured as a function of time at 160 °C, 180 °C and 200 °C: (**a**) pure BA-ph and (**b**) BA-ph/PEN blends with 20 wt % PEN content.

The curing behaviors of the BA-ph and the BA-ph/PEN blends with various contents of PEN were studied by DSC and DRA. The curves are presented in Figure 1 and Figure 2. Also, the Table 1 lists the main data of the copolymerization process. The Figure 1a corresponds to the DSC curves of the blends and Figure 1b to the composites cured at 180 °C/2 h and 200 °C/2 h. It can be seen from Figure 1a that the DSC curve of PEN exhibits a clear glass transition temperature (*T_g_* = 178 °C). The various BA-ph/PEN blends display obvious two-step curing processes, a gentle exothermic peak (marked as A) and a sharp exothermic peak (marked as B). The peak A at the temperature 226–233 °C can be assigned to the ring-opening reactions of benzoxazine rings, thus to form the Mannich bridge structures and generate the phenolic hydroxyl groups. The peak B at the temperature 268–275 °C is related to the ring-forming polymerization of nitrile groups [30,31]. It can be seen that the initial curing temperature (at around 168 °C) and the temperature of two exothermic peaks slightly shift to the higher temperature with the increasing PEN content. This can be ascribed to that before the crosslinking network structure formed, the viscosity increases with the incorporation of the polymer PEN that decreases the motivation and interaction of the functional groups, thus to retard the polymerization of BA-ph/PEN to some extent [32]. What’s more, the PEN molecular acts as the diluent that decreases the density of the functional groups and increases the distance between the adjacent functional groups, leading to the lower polymerization degree of the blends at the higher curing temperature [15]. Compared to the pristine BA-ph, the *ΔH_curing1_* (ring-opening reactions of benzoxazine rings) of the blends does not significantly changed, while, the *ΔH_curing2_* (ring-forming polymerization of nitrile groups) rapidly decreases as the PEN content increases (≥20 wt %). From the results of the DSC, it is obvious that PEN shows few effects on the ring-opening polymerization of oxazine rings, while showing observed inhibition on the ring-forming polymerization of nitrile groups. It is well known that the nitrile groups in PEN are hard to polymerization due to its sluggishness itself, as well as the inhibition of the long molecular chains. Thus, with increasing the content of PEN, the polymerization enthalpy of nitrile groups presented in Table 1 (*ΔH_curing2_*) decreases. However, when the PEN content increases to 40 wt %, the decrease trend of the polymerization enthalpy of nitrile groups weakens with increasing PEN content. It can be assigned to the fact that the nitrile groups in PEN also participated into the polymerization.

As shown in Figure 1b, the two exothermic peaks almost disappear after being treated at 180 °C/2 h and 200 °C/2 h for all of the blends. It indicates that the ring-opening polymerization of oxazine rings and the ring-forming polymerization of nitrile groups were completed at this condition. In comparison with that of previous reports, the complete polymerization of nitrile groups at 200 °C indicates the outstanding process properties of BA-ph. Moreover, due to the active hydrogen and amine structures generated from the ring-opening of oxazine, the copolymerization between PEN and BA-ph occurs in this work.

**Scheme 1:**
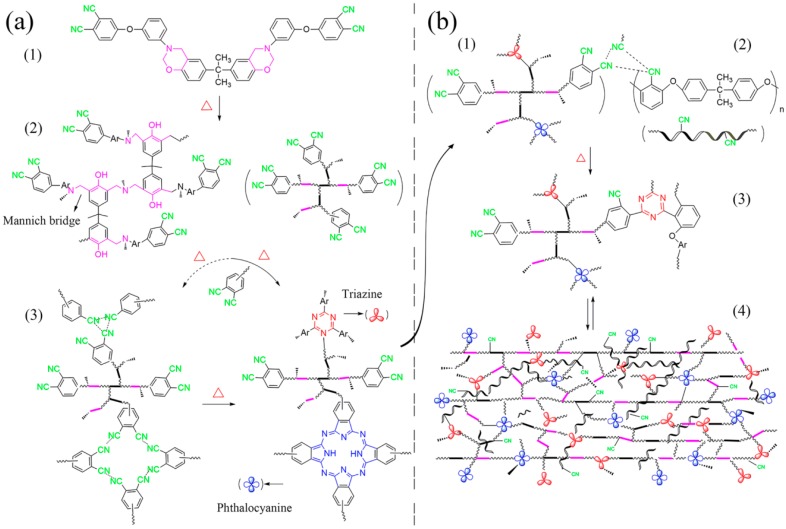
(**a**): (1) the structure of the BA-ph, (2) the structure of Mannich bridge formed from the ring-opening of benzoxazine rings, (3) the structure of phthalocyanine and triazine rings generated from the ring-forming polymerization of the nitrile groups; (**b**): (1) the simplified structure of cured BA-ph, (2) the structure of PEN, (3) the structure of triazine ring derived from the copolymerization of nitrile groups between BA-ph/PEN matrix and EPN, (4) the possible structure of the matrix resins.

Dynamic rheological analysis was applied to study the curing behavior and processability of BA-ph and BA-ph/PEN blends. The viscosity (η^*^) change of BA-ph (Figure 2a) and BA-ph/PEN (Figure 2b) blends with the 20 wt % PEN content was measured as a function of time at 160, 180 and 200 °C, respectively. As shown in Figure 2, it can be seen that the viscosity of the BA-ph and BA-ph/PEN blends increased with increasing the temperature. The viscosity is stable and low before the curing reaction starts. The viscosity of the two systems is greatly influenced by the high temperature, because of that the higher temperature the faster the curing reaction occurs. It is obvious that it takes about 25, 5 and 1 min until the reactions occur at 160, 180 and 200 °C, respectively. In Figure 2b, it takes about 32, 9 and 2 min until the reaction occurs at the same temperatures, indicating that with the introduction of PEN, the polymerization rate of the matrix decreases, which is in well agreement with the results of DSC. Before the curing reaction intensively carried out, the viscosity of the BA-ph and BA-ph/PEN matrix were lower than 100 Pa·s which exhibited a good flowability. The good flowability of matrix is crucial for the preparation of the composites in the operation. The good flowability of the matrix can contribute to the good interlaminar adhesion and the smooth surface of the composites. Additionally, a proper operation time (about 5–10 min) should be needed in the operation. So, the temperature 180 °C was chosen as the initial processing temperature. As for the temperature 200 °C, the polymerization of the matrix carried out intensively in a short time (<1 min) and the viscosity rapidly increased in a relatively short time. And there was no time left for the operation. Thus, considering of the processability, 180 °C can be selected as the proper process temperature. The possible curing processes of BA-ph/PEN were presented in Scheme 1.

### 3.2. Mechanical Properties of the p-GF-Reinforced Composites

**Figure 3:**
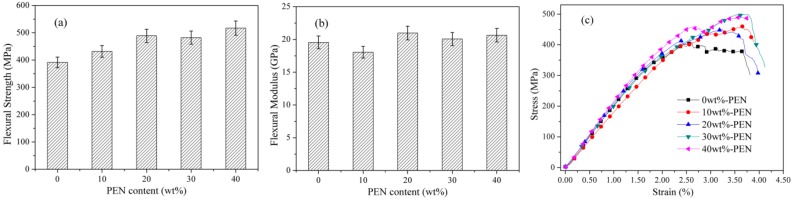
Mechanical properties of the various composite laminates: (**a**) the flexural strength, (**b**) the flexural modulus and (**c**) stress and strain curves.

According to the results of the curing behaviors and process properties, the fiber-reinforced BA-ph/PEN composite laminates have been successfully fabricated. Mechanical properties were investigated to furthermore confirm the effects of modification of the GFs. The effects of PEN introduced into the BA-ph matrix on the flexural strength and modulus of the composites were also studied and shown in Figure 3. As shown in Figure 3a, the flexural strength of composites with various contents of PEN (0, 10, 20, 30, 40 wt %) is 391.45, 431.87 (increased by 10.3%), 488.67 (increased by 24.8%), 482.07 (increased by 23.1%) and 517.22 (increased by 32.1%) MPa, respectively. With increasing the content of PEN, the flexural strength increases correspondingly. It is well known, the flexural strength of the laminates is dominated by the matrix and the interfacial adhesion between the matrix and fibers. In this work, on the one hand, the improvement can be attributed to the flexible PEN molecular chains that can greatly increase the toughness of the BA-ph matrix [33]; on the other hand, the PEN chains decorated on the surface of the GFs also can roughen the surface, which would contribute to improve the interface interaction between fibers and the matrix.

Figure 3b presented the flexural modulus of the composite laminates with various content of PEN in the matrix. The flexural modulus value of 19.53, 18.05 (decreased by 7.5%), 20.97 (increased by 7.3%), 20.06 (increased by 2.7%) and 20.63 (increased by 5.6%) GPa is assigning to 0, 10, 20, 30 and 40 wt % of PEN content. It is clearly that the flexural modulus decreases while the composites with 10 wt % PEN, then increases with increasing the content of PEN. When the PEN content reaches to 20 wt %, the flexural modulus reaches to the maximum. The decreasing of the modulus can be explained with the fact that the thermoplastic polymer PEN acts as the plasticizer which will reduce the interaction between the rigid crosslinking networks. Then, the flexural modulus increases with the PEN content increased, this is due to the copolymerization of BA-ph and PEN that forms the semi-interpenetrating polymer networks [34]. In the discussion of DSC and DRA, the results also indicate that the copolymerization occurred between BA-ph and PEN when the content of PEN over to 20 wt %. Considering of the modulus of the PEN and BA-ph polymers themselves, the thermosetting PEN shows a relative low modulus. Thus, persistently increasing the content of PEN (40 wt %), the flexural modulus of the composites will present a weak increase, due to the fact that the flexible PEN chains may dominate the flexural modulus. Besides, the composite with 20 wt % PEN shows the highest flexural modulus, which is also ascribed to the surface modification. The better interfacial interaction would contribute to the higher mechanical properties [18,24]. The GFs coated with PEN increased the wettability between the GFs and the matrix. What’s more, the copolymerization between the PEN and the BA-ph/PEN matrix resin occurs on the surface of GFs. In addition, the stress and strain curves were presented in Figure 3c. In sum, the modification of GFs and the BA-ph matrix with PEN simultaneously, can obviously improve the mechanical properties, including the flexural strength and modulus.

### 3.3. Fracture Morphology of the p-GF-Reinforced Composites

**Figure 4:**
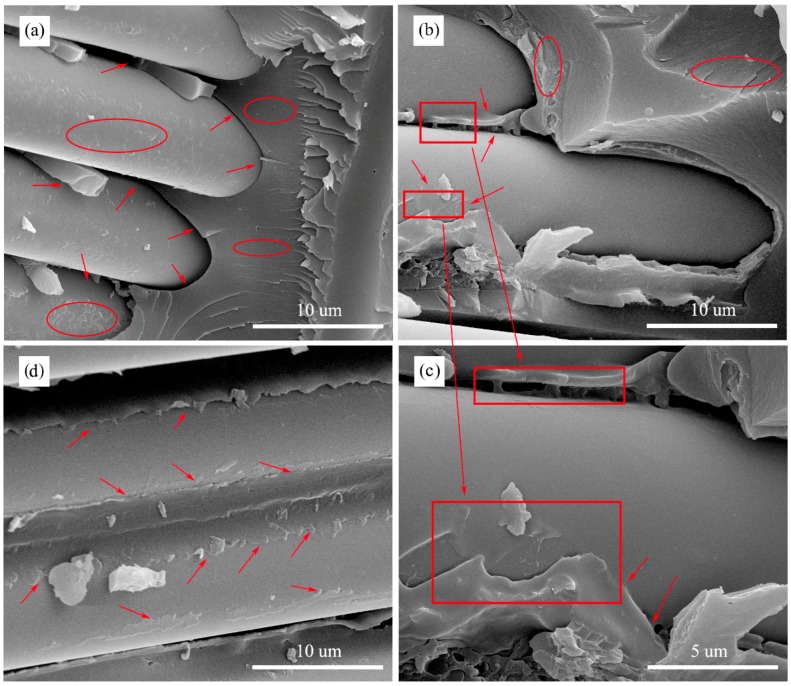
SEM image of the various composite laminates: (**a**) BA-ph; (**b**) BA-ph/PEN blends with 20 wt % PEN content; (**c**) the zoom in image of (**b**,**d**) BA-ph/PEN blends with 40 wt % PEN content; the arrows reflected the gaps between the matrix and GF in Figure 3a, as well as adhesion properties in Figure 3b–d; the ovals reflected the rough surface of GF and the smooth surface of the matrix; the rectangles marked the zoom in image of the adhesion properties of the composites in a particular area.

To further study the interface interaction and interfacial compatibility, SEM images of the fracture surface of the composites were investigated and shown in Figure 4. Figure 4a,b,d presented the surface of the composites with 0, 20 and 40 wt % PEN. Figure 4c was the zoom in image of b. It can be seen in Figure 4a that fish-scale can be obviously observed on the surface of the GFs. The fish-scale is attributed to the intertwining and accumulation of PEN chains. Although the decorated GFs possess a rough surface, the obvious cracks between the GFs and the BA-ph matrix is observed. It indicates the interfacial compatibility of the BA-ph matrix and the decorated surface is not improved intrinsically. The fracture morphology of the BA-ph matrix resin is smooth and the matrix debris exists in the gap without any adhesion to the GFs, revealing that the BA-ph matrix showed the brittleness properties.

Figure 4b shows the fracture surface of the composites with 20 wt % PEN in the BA-ph matrix. No fish-scale can be observed on the surface of the GFs and no cracks are observed in sight, indicating the improved interfacial compatibility between the fibers and the matrix. Moreover, the GFs are coated with the BA-ph/PEN matrix, showing an obvious interface layer. The adhesion effects of the BA-ph/PEN matrix are also observed between the fibers. Figure 4c presents the zoom in image of Figure 4b. It can be seen that the coated matrix on the surface of the fibers and the adhesion effects of the BA-ph/PEN matrix are significant. As for the BA-ph/PEN matrix with 20 wt % PEN, the fracture surface is rough and irregular, which means that the toughness of BA-ph is improved by incorporation of PEN. Figure 4d presents the fracture surface of the composites with 40 wt % PEN, the image exhibits good interfacial adhesion between the GF and matrix. No pulled out GFs are observed and the GFs are all embedded in the BA-ph/PEN matrix, which indicating that the interfacial compatibility of the GFs and matrix is improved. In sum, the GFs modified with PEN before being infused the prepolymers to prepared the composite laminates could significantly roughen the surface, thus to improve the interface properties of the composites.

**Scheme 2:**
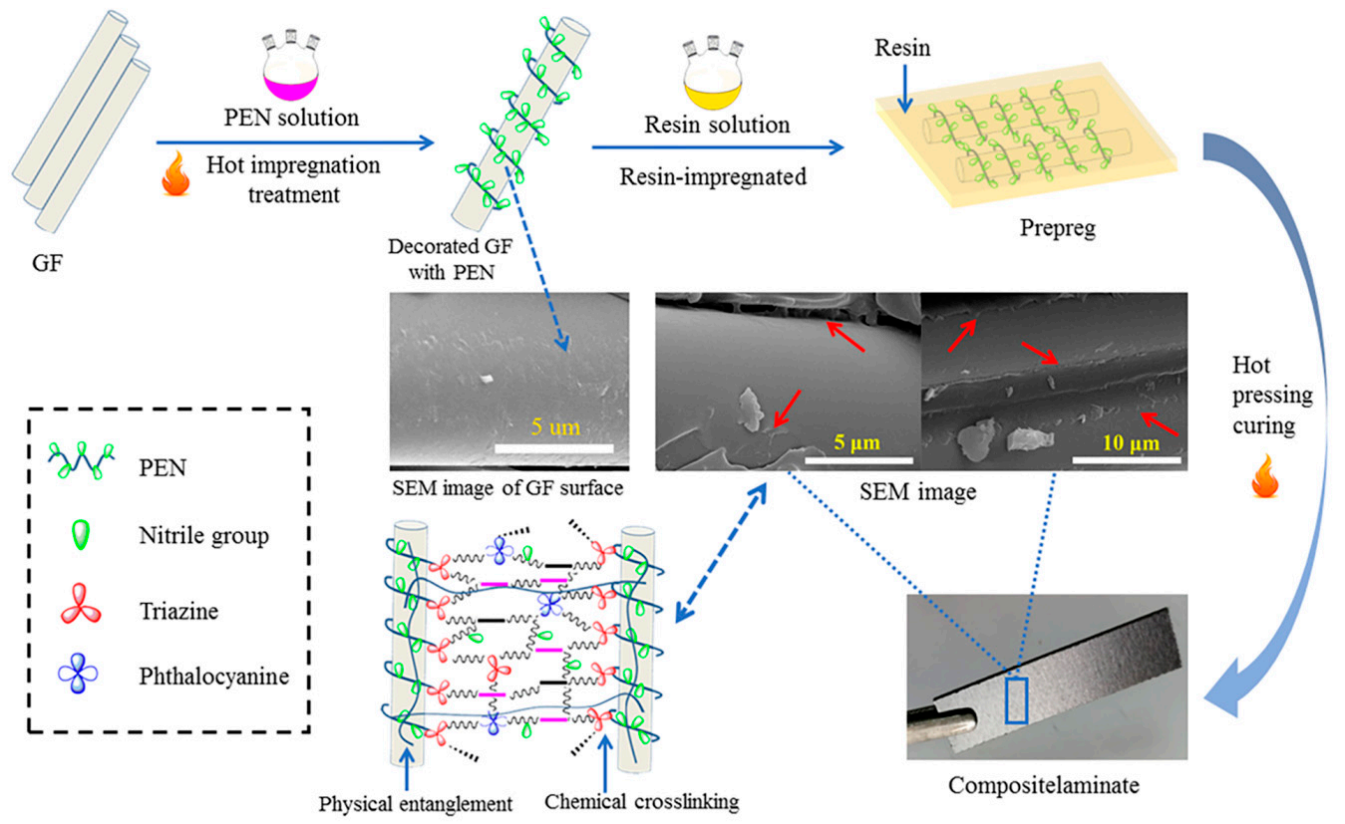
The possible mechanism of the improved interfacial adhesion of the composite laminates.

Moreover, the interfacial adhesion displays an obvious difference between the matrix resin with existence or not of PEN. The matrix with PEN shows better interfacial adhesion than the pristine BA-ph/*p*-GF composite. This can be attributed to the possible facts as follows: (1) the entanglement of the long molecular chains between the PEN on the surface of GFs and in the matrix; (2) the copolymerization of nitrile groups that from PEN on the surface of GFs and from the matrix. For the BA-ph/*p*-GF composites, due to the high rigidity and brittleness of the thermosetting BA-ph, the entanglement between the molecular chains is difficult. What’s more, the copolymerization between the BA-ph and PEN is difficult to occur due to the self-polymerization of BA-ph. Besides, the brittle interface layers forms mainly from the polymerization of BA-ph, will be easily destroyed under the pressure. For the matrix with PEN, BA-ph/PEN matrix possesses good compatibility with GFs due to the possible copolymerization mentioned above, as well as the physical entanglement between BA-ph/PEN blends and the PEN on the surface of GFs. Moreover, the toughness of the matrix is improved with the introduction of PEN. It’s beneficial to absorb more energy and hinder the crack generation to some extent. In other words, the properties can be improved though modifying the GFs with a high performance thermoplastic polymer and then incorporating the thermoplastic polymer into the thermosetting matrix, simultaneously. The possible enhanced mechanisms of the improved interface compatibility between the decorated GFs and the BA-ph/PEN matrix are presented in Scheme 2.

### 3.4. Dynamic Thermomechanical Analysis of the p-GF-Reinforced Composites

**Figure 5:**
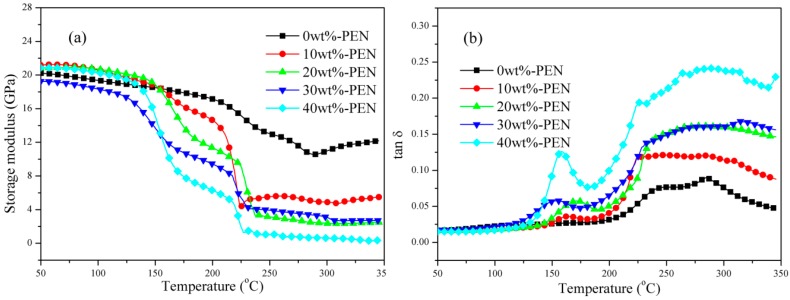
Dynamic mechanical analysis curves of the various composite laminates: (**a**) storage modulus and (**b**) *tan δ.*

Dynamic mechanical studies were applied to study the storage modulus and the loss tangent of the composites. Figure 5a presented the storage modulus of the various composites as a function of temperature in range 50–350 °C. It indicates that the BA-ph/PEN matrix possesses the similar rigidity. As can be seen the initial storage modulus of the composites at 50 °C increased with the incorporation of PEN, which was well in coincidence with the change of flexural modulus. So, at the lower temperature the mechanical properties of the composites were improved by adopting PEN. With increasing the content of PEN, the rigidity of the matrix decreases slightly, due to the flexible PEN chains. With increasing the test temperature, the storage modulus of the composites with PEN slightly decreases at about 150 to 210 °C and then sharply decreases at 225 °C. A platform can be observed for all of the composites at above 225 °C. It can be explained by the fact that the flexible PEN chains contribute to the decreasing of the rigidity of the matrix. When the temperature went on, the rigidity of the composites decreased faster than the composites without the PEN. As for the BA-ph/*p*-GF composite, the storage modulus decreases rapidly after 200 °C and reaches the minimum after 280 °C. This can be attributed to the fact that the BA-ph can form the rigid Mannich bridge structures and heteroaromatic rings including the phthalocyanine and triazine rings, thus to exhibit an outstanding properties at high temperatures. For the BA-ph/PEN/*p*-GF composites, the fact that the PEN acts as plasticizer in the matrix and the movements of the PEN molecular chains at glass transition temperature (*T_g_* = 178 °C, tested by DSC shown in Figure 1) will cause the storage modulus decrease earlier than that of the pristine BA-ph/*p*-GF composites.

The plots of *tan δ* of the *p*-GF-reinforced composites were presented in Figure 5b. For the fiber-reinforced matrix composites, the *tan δ* can reflect the mechanical damping effects of the composites. It is reported that the loss transitions appeared at 100–150 °C are assigned to the secondary relaxation of the molecular chains [35,36]. Then, the wide and high loss bands for all of the composites appear and could be attributed to the glass transition process. Two transitions at 225 and 280 °C are observed. The transitions can be assigned to the Mannich bridge structures and the heteroaromatic structure generated from the ring-forming polymerization of the nitrile groups. Moreover, the obvious transition processes also can be attributed to the semi-interpenetrating networks formed by the PEN chains embedded into the BA-ph polymers. With increasing the content of PEN, the height of the loss factors increased. This is due to that with the incorporation of the PEN, the rigidity of the crosslinking network decreases and the movement of molecular chains of the matrix resins increase. As we all know, the matrix resins with more flexibility will lead to more energy dissipate, that possess greater damping [18,37]. In sum, the introduction of PEN in the BA-ph matrix can improve the interfacial compatibility of the BA-ph/PEN/*p*-GF composites, meanwhile, increasing the damping of the composites, that would enable them to be applied as the seismic materials with outstanding modulus and thermal-mechanical properties.

### 3.5. Thermal Properties of the p-GF-Reinforced Composites

**Figure 6:**
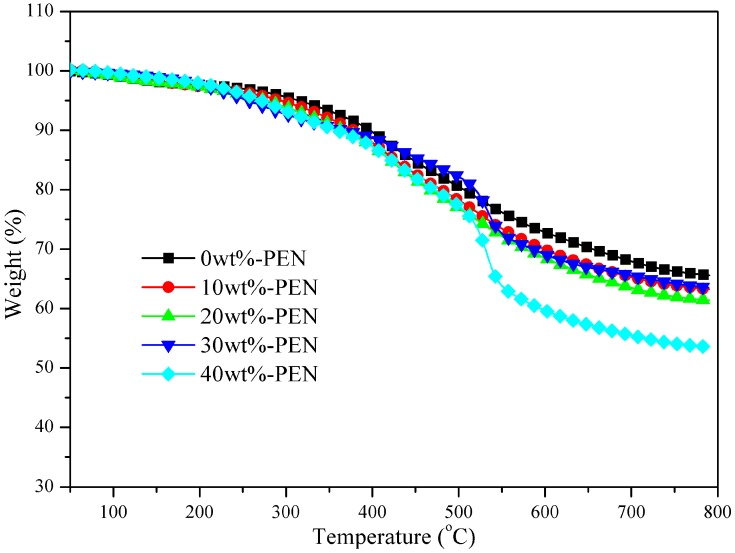
Thermal properties of the various composite laminates.

Thermal properties of the BA-ph/*p*-GF and BA-ph/PEN/*p*-GF composites were studied by TGA under nitrogen atmosphere with the heating rate of 20 °C/min from 50 to 800 °C. The curves were shown in Figure 6 and the main data was listed in Table 2, including the weight loss of 5 wt % (*T_5%_*), 10 wt % (*T_10%_*) and the char yield at 800 °C. The thermal stability of the composites can be evaluated by the *T_5%_*. As for PEN, the *T_5%_* is about 490 °C, exhibiting outstanding thermal stability [26]. From the curves of TGA and the data listed in Table 2, the decomposition temperatures of *T_5%_* and the char yield for the composite laminates with 10% and 20% PEN gradually decrease with increasing the content of PEN. This is due to the fact that the PEN could hinder the polymerization of the BA-ph. The results well agree with the results of DSC testing and dynamic rheological analysis. As for the 30 wt % and 40 wt % PEN content, because of the relative high PEN content, a rapid decomposition happens at 500–550 °C which can be corresponded to the low crosslinking degree of the matrix resin with high content of PEN. In sum, the composites prepared under the current curing procedure (200 °C) could exhibit good thermal stability.

## 4. Conclusions

In this work, the decorated GFs with PEN were designed and their reinforced matrix (BA-ph/PEN) composites were prepared at relative low process temperature (200 °C). The curing behavior and dynamic rheological of BA-ph/PEN were studied, results indicated that the PEN existed in the matrix would hinder the curing reaction of the BA-ph. However, with introduction and increasing the content of PEN into the BA-ph matrix, the mechanical properties of the BA-ph/PEN/*p*-GF composites were improved, which could be explained by the improvement of the matrix and the enhancement of the interfacial adhesion. Combined the results of DSC and DRA, semi-interpenetrating polymer networks may be formed during the copolymerization between PEN and BA-ph, which would improve the toughness of the matrix. Moreover, the PEN decorated on the GFs formed fish-scale on the surface of the GFs can significantly roughen the surface. The rough surface would significantly improve the interface compatibility through the entanglement of the molecular chains and the copolymerization of the active nitrile groups. The proposed hypothesis was confirmed with the investigation of the fracture surface and the thermal-mechanical properties. In this work, it’s proposed that a convenient and efficient method to modify the brittleness and improve the interfacial interaction of the fiber-reinforced composites to obtain the composite laminates with improved properties. In our further study, the investigation of the formation of the interpenetrating polymer networks between the PEN and BA-ph polymers will be carried out in the successive works.

## Figures and Tables

**Table 1:** Thermal properties of the various BA-ph/PEN blends.

	*T_g_* (°C)	*T_i_* (°C)	*T_top1_* (°C)	*T_top2_* (°C)	*ΔH_curing1_* (J/g)	*ΔH_curing2_* (J/g)
BA-ph	-	166	226	268	61.22	60.11
10 wt %	-	168	227	269	61.73	52.67
20 wt %	-	168	230	270	69.64	35.32
30 wt %	-	181	232	274	45.27	17.91
40 wt %	-	181	233	275	51.02	13.77
PEN	178	-	-	-	-	-

**Table 2:** Thermal properties of the various composite laminates.

	0 wt %	10 wt %	20 wt %	30 wt %	40 wt %
*T_5%_* (°C)	315.14	293.94	276.41	256.17	270.79
*T_10%_* (°C)	397.32	377.96	367.01	367.7	358.43
Char yield (%)	65.61	63.27	61.41	63.51	53.53
